# Leveraging Optimized Transcriptomic and Personalized Stem Cell Technologies to Better Understand Syncytialization Defects in Preeclampsia

**DOI:** 10.3389/fgene.2022.872818

**Published:** 2022-03-30

**Authors:** Sehee Choi, Teka Khan, R. Michael Roberts, Danny J. Schust

**Affiliations:** ^1^ Department of Obstetrics, Gynecology and Women’s Health, University of Missouri School of Medicine, Columbia, MO, United States; ^2^ Christopher S Bond Life Sciences Center, University of Missouri, Columbia, MO, United States; ^3^ Division of Animal Sciences, University of Missouri, Columbia, MO, United States

**Keywords:** syncytiotrophoblast, single nuclei RNA sequencing, trophoblast, preeclampsia, stem cell, trophoblast models

## Abstract

Understanding the process of human placentation is important to the development of strategies for treatment of pregnancy complications. Several animal and *in vitro* human model systems for the general study human placentation have been used. The field has expanded rapidly over the past decades to include stem cell-derived approaches that mimic preclinical placental development, and these stem cell-based models have allowed us to better address the physiology and pathophysiology of normal and compromised trophoblast (TB) sublineage development. The application of transcriptomic approaches to these models has uncovered limitations that arise when studying the distinctive characteristics of the large and fragile multinucleated syncytiotrophoblast (STB), which plays a key role in fetal-maternal communication during pregnancy. The extension of these technologies to induced pluripotent stem cells (iPSCs) is just now being reported and will allow, for the first time, a reproducible and robust approach to the study of the developmental underpinnings of late-manifesting diseases such as preeclampsia (PE) and intrauterine growth retardation in a manner that is patient- and disease-specific. Here, we will first focus on the application of various RNA-seq technologies to TB, prior limitations in fully accessing the STB transcriptome, and recent leveraging of single nuclei RNA sequencing (snRNA-seq) technology to improve our understanding of the STB transcriptome. Next, we will discuss new stem-cell derived models that allow for disease- and patient-specific study of pregnancy disorders, with a focus on the study of STB developmental abnormalities in PE that combine snRNA-seq approaches and these new *in vitro* models.

## Introduction

The human placenta is unique among eutherian mammals, and some of its morphological features, such as its markedly deep level of placental cell [trophoblast (TB)] invasion into maternal tissues, are inextricably linked to characteristically human pregnancy disorders such as preeclampsia (Pennington et al., 2012; Khong et al., 2015; Windsperger et al., 2017; Zhou et al., 2021). There are several sublineages of TB present in the placenta. The very large and multinucleated syncytiotrophoblast (STB) layer is one of the three main sublineages of placental TB in humans and other species with a hemochorial type of placentation. The others are the invasive extravillous TB cells (EVT) and the proliferative mononucleated cytotrophoblast cells (CTB), although this classification is undoubtedly an oversimplification and further definable subpopulations undoubtedly exist within each of the three large groupings. For example, there are several described functional subcategories of the CTB in the anchoring villi of the human placenta as they near the maternal decidua that likely correlate with their differentiation into pre-EVT cells (Tsang et al., 2017; Pollheimer et al., 2018; Shannon et al., 2022). The CTB of the villi proper show changes as they prepare for syncytialization into STB and some CTB subpopulations appear to remain in as proliferative stem-cell like progenitors (Handwerger, 2010). The EVT sublineage is further divided into interstitial EVT that invades through the maternal decidua, where they interact directly with maternal decidual immune cells, endoglandular EVT when they are found in the walls of decidual glands and endovascular EVT when they replace the endothelium and surrounding layers of the uterine spiral arteries (Moser et al., 2017; Huppertz, 2019). Aberrations in the development and function of each of the TB subpopulations will likely cause disorders of pregnancy and teasing out the normal and abnormal developmental pathways of each and how they interact with each other and with surrounding maternal cells is a lofty but now attainable goal. In this review, we will describe our approach to the *in vitro* study of STB transcriptomics using single nuclei RNA sequencing (snRNA-seq) that can be applied to stem cell-derived and primary TB models. We will also discuss recent and ongoing work that uses stem cell-derived *in vitro* TB models to incorporate personalized approaches into the study of disorders of human pregnancy and the use of snRNA-seq in these models to better define STB-specific defects.

## Next-Generation Sequencing-Based Approaches to the Study of Human Syncytiotrophoblast

In the first section of this review, we will focus on the STB sublineage. The STB, the critical maternal-fetal exchange interface, is established from the terminal differentiation and fusion of CTB (Gude et al., 2004; Poidatz et al., 2015). There are three types of STB that arise during different stages of human gestation. The first is a primitive STB that originates during early pregnancy (second week after fertilization) and facilitates fetal implantation by decidual erosion. The second is villous STB, which lines the exterior of the chorionic villi. While primary chorionic villi emerge at approximately the third week of gestation (post-fertilization) and exist throughout the remainder of gestation, the earliest stage for the detection of villous STB is still not determined (Jaremek et al., 2021). The third form of syncytialized TB is the little understood and poorly studied giant cells. So little is known about the origins of the latter, that this STB sub-type will not be discussed further here. As to whether or not the primitive STB of the implanting embryo and villous STB are related ontologically and represent the progressive evolution of the same lineage as gestation progresses or whether they are distinct entities is still debatable (Jaremek et al., 2021).

Villous STB comprises the outermost layer of the human placenta and is hence in direct contact with maternal peripheral blood. STB represents 70% of all cells present in placenta and forms a continuous layer that covers the entirety of the extensive surface of the chorionic villi (Blackburn et al., 1988; Fuchs and Ellinger, 2004). Being the continuous outermost layer of the human placenta, STB is in direct contact with circulating maternal blood and thereby acts as physical barrier to restrict transmission of infection from mother to fetus (Ander et al., 2018). While many mechanisms for placental resistance to viral infection are known, the STB lacks the cell junctions and receptors commonly used by many viruses for cell entry so that even the morphology of this barrier aids in this defense. STB secretory products, such as progesterone and chorionic gonadotropin, and particles of various sizes, including microparticles, microvesicles and syncytial knots, are released into the maternal circulation. These secretory products modulate maternal physiology but can also have diagnostic potential for fetal-placental aberrations and other disorders of pregnancy (Martinez et al., 2015; Jaremek et al., 2021). The latter term, syncytial knots, was first described by Tenney and Parker (Tenney Jr & Parker Jr, 1940) and is a general term used for the heterogeneous group of STB aggregates that include syncytial bridges, syncytial sprouts, syncytial clumps, proliferation knots, nuclear clumps, trophoblastic extensions, and apoptotic knots and are continuously shed into the maternal circulation, since STB undergoes apoptosis and necrosis readily (Bainbridge et al., 2006; Loukeris et al., 2010). Some have reported that the presence of syncytial knots in more than 30% of terminal villi is consistent with pregnancy disorders of abnormal placental form and function, such as preeclampsia (PE) (Loukeris et al., 2010). As the outermost selectively permeable layer of the placenta, STB also serves in a buffering capacity, protecting the fetus from the harmful compounds circulating in mother’s blood. Despite the importance of STB formation and function for fetal development and pregnancy outcomes, limitations in cellular and animal models have left much to be discovered (Jaremek et al., 2021).

The availability of RNA-seq, a next generation sequencing technology, has provided an unprecedented opportunity for researchers and clinicians to analyze single cell and tissue transcriptomes at a far higher resolution than previously used technologies such as transcript cloning with subsequent Sanger sequencing and microarray (Martinez et al., 2015). Briefly, with RNA-seq technologies, complementary DNAs (cDNAs) are generated from RNAs of interest and subsequently aligned to a reference genome for the construction of whole-genome transcriptome maps (Nagalakshmi et al., 2010). RNA-seq data analyses have generated deeply accurate and consistent results, leading to the discovery of new isoforms and variants of genes in a number of mammalian tissues and organs (Nagalakshmi et al., 2010). Further, RNA-Seq has proved very successful in quantifying transcript levels precisely, confirming or revising previously annotated 5′ and 3′ ends of genes, and mapping exon/intron boundaries (Nagalakshmi et al., 2010). Application of RNA-seq has markedly enhanced our understanding of gene expression complexity and gene regulation in placental tissue and elsewhere.

Investigation of cellular activity, state, and physiology requires rapid genome and transcriptome analysis technologies. Beginning with a low-throughput technique called Northern blot analysis (Alwine et al., 1977), the first candidate gene-based approach, progressive advancements have been made toward the analysis of cellular dynamics that inform our understanding of cell physiology and fate. These general approaches have been extensively reviewed (Morozova et al., 2009) (Mutz et al., 2013), including very recent publications that focus on the placenta and even specifically on STB (Yong & Chan, 2020) (Jaremek et al., 2021). Depending on the experimental questions, these various approaches to sequencing can be classified into three major categories: 1) candidate gene approaches such as reverse transcription quantitative PCR (RT-qPCR) and Northern blot, 2) microarray, a high-throughput gene expression profiling tool involving hybridization of mRNA to an array of complementary DNA probes corresponding to genes of interest, and 3) RNA-seq (Morozova et al., 2009; Yong & Chan, 2020). RNA-seq is a more recent and deep sequencing technology than microarray that can generates a fuller picture of the cell/tissue/organ transcriptome (Yong & Chan, 2020). In brief, a library of cDNA fragments is constructed from adapters (usually poly A) attached to one or both ends of the RNA (total or fractionated). Next, cDNAs with or without amplification, are sequenced in a high-throughput manner to obtain 30–400 bp reads from one end (single-end sequencing) or both ends (pair-end sequencing) (Wang et al., 2009). The generated data are usually in gigabyte amounts and require a well-organized pipeline to be analyzed. Typically, the RNA-seq analysis pipeline is comprised of the following fundamental steps. The sequence data are first aligned with a reference genome or transcriptome representing the organism being studied. The mapped reads are counted, and gene expression level is estimated by peak calling algorithms. Ultimately, differential gene expression is determined by using statistical analyses. (Mutz et al., 2013). RNA-seq has the ability to identify single-nucleotide variants in both coding and non-coding RNA (all lengths), determine rare and novel transcripts, and is not species dependent. Moreover, recent developments in RNA-seq technology enables the establishment of transcriptome profiles either in individual cells (scRNA-seq) or individual nuclei (snRNA-seq) (Hu et al., 2018).

### RNA-Seq for *In Vivo-* and *In Vitro*-Generated TB and Difficulties in Syncytiotrophoblast Transcriptome Profiling

The multi-functional placenta is central to pregnancy outcomes and the subsequent health of both the mother and her child, so that it is not surprising that genome-wide profiles of primary placental tissues and cells and *in vitro* TB models have been studied extensively. As reviewed by Yong and Chan (Yong & Chan, 2020), RNA profiles of normal placental samples obtained across gestation provide an opportunity to understand healthy development and can act as a reference to see how the placenta responds and adapts to various exposures and challenges in complicated pregnancies. Similarly, profiling the placental transcriptomes from compromised pregnancies helps to identify pathological changes associated with specific clinical phenotypes and can be subsequently expanded to allow for the development of diagnostic biomarkers and therapeutic interventions. Pregnancy is a markedly dynamic state, so that placental gene expression profiles that reflect such phenomena as regulation of fetal growth, immunological tolerance, and physiological changes that accommodate the changing needs of both mother and fetus will also be dynamic. This highlights the utility of a combination of bulk tissue level RNA-seq and sc/snRNA-seq technologies to fully reflect the heterogeneity of cell and molecular interactions across the full spectrum of gestational stages. Here, we will focus on existing studies that employ scRNA-seq on the human placenta, since they have identified and highlighted the technical challenges presented by a large syncytialized and multinucleated STB for RNA-seq analyses and have led us to explore the utility of snRNA-seq to address these limitations.

A number of studies have been conducted on placental samples obtained from normal, preeclampsia or preterm birth conditions that cover gestational ages from 6 weeks (wks) to full term (as reviewed by H. Li et al., 2020) (Li et al., 2020). Suryawanshi et al. performed scRNA-seq from freshly-collected first trimester (6–11 weeks of gestation) chorionic villi (*n* = 8) and matched maternal decidual (*n* = 6) samples (Suryawanshi et al., 2018). Using data from samples of placental villi, they were able to describe the known TB subtypes (i.e., CTB, EVT, STB) and cell populations from the villous cores that included Hofbauer cells, endothelial cells, and mesenchymal stromal cells. Analyses of the proliferation markers *MKI67*, *TOP2A*, *TK1*, and *PCNA* identified subpopulations of proliferating and non-proliferating cells among CTB and mesenchymal stromal (aka fibroblast) cells, although the authors did not specifically describe how these subpopulations differed from one another nor their potential functions (Suryawanshi et al., 2018). Y. Liu et al. carried out scRNA-seq analysis generated from 8 weeks villous and 24 weeks decidual cells from human placental samples and distinguished 14 distinct placental cell subtypes (Liu et al., 2018). In their villous transcriptome analysis, they identified three CTB subtypes, two EVT subtypes, two *CD90*, *ENG* and *CD74* tri-positive mesenchymal stromal cell subtypes, two *CD68*-positive Hofbauer cell subtypes, and an STB population (Liu et al., 2018). Tsang et al. used scRNA-seq to define individual cell populations in the human term placenta and reconstructed a TB differentiation trajectory from cell subgroups (Tsang et al., 2017). The importance of this work lies in its identification and description of the multiple cellular subtypes, their organization and functions, and the inferred molecular interactions within and among the identified cells. The description of the STB component of their analyses, however, is extremely limited in comparison. For instance, among the three types of CTB, one seems to be more proliferative as suggested by differentially higher expression of cell cycle signatures that include *RRM2*, *CCNB1* and *CDK1*. Another CTB subtype with the lowest proliferative profile, is identified to be the one with the greatest profusogenic potential since it has maximal expression of placental fusogens such *as ERVFRD-1* (*Syncytin-2*). This CTB subtype may be the population from which the villous STB emerges. Beyond this, however, the authors provide limited information on other TB subpopulations, and no detailed transcriptomic information for the STB. This leaves the reader to question whether the STB may also have subtypes and, if so, how these subtypes might differ. The various authors did, however, leverage their placental scRNA-seq data to predict patterns of virtual differentiation by pseudo time analysis and thereby describe a patterned progression from progenitor CTB and ending on well differentiated EVT. (Liu et al., 2018; Shannon et al., 2022). Further, the expression across gestation of transcription factors (TFs) regulating the differentiation from CTB to EVT, including *ZNF124, SOX4, ACL2, ZNF555*, was also analyzed [Figures 3D,E from Liu et al. (Liu et al., 2018)]. Unfortunately, but likely reflecting the limitations of scRNA-seq in the placenta, the differentiated STB is absent at all from the pseudo time analysis and the TFs involved in the emergence of STB from CTB have been neither identified nor reported.

**FIGURE 1:**
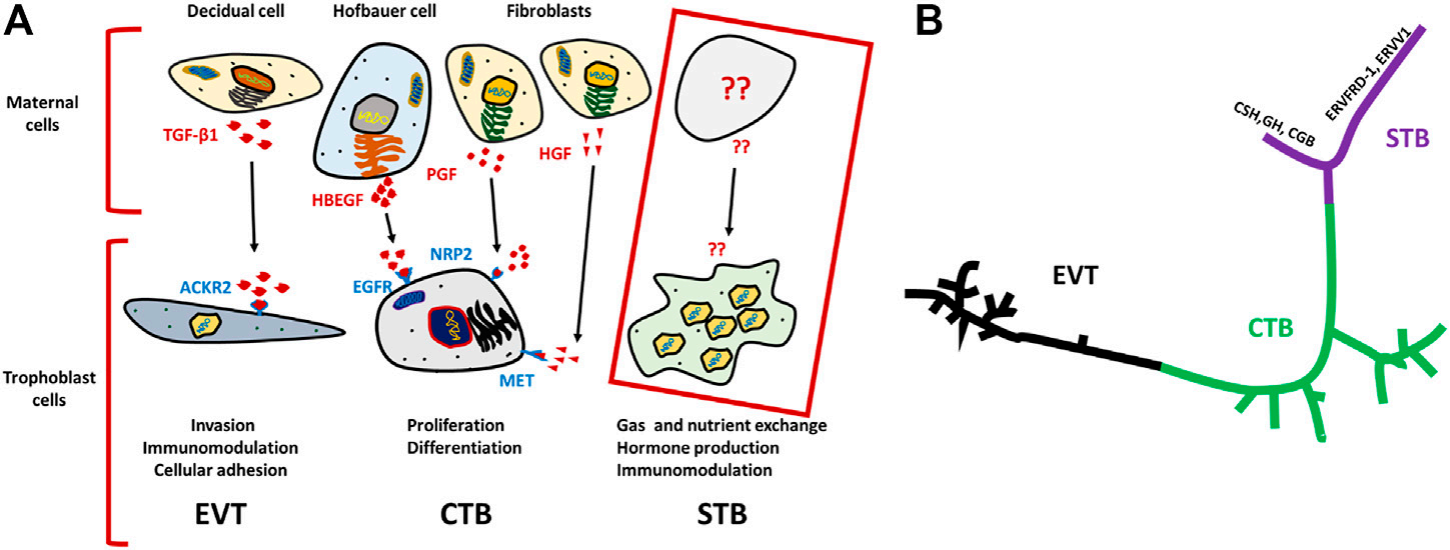
Schematics for ligand–receptor pairs and Pseudo time analysis of TB cells. **(A)** Ligands (indicated in red) are released by various maternal cells, of which predicted receptors are present on respective TB cells (indicated in purple). TGF-β1 ligands for ACKR2 of EVT cells, is released by decidual cells and predicted to trigger EVT invasion, immunomodulation and cellular adhesion. Similarly, EGFR present on CTB potentially interacts with HBEGF released by Hofbauer cell, and PGF and HGF ligands are secreted from fibroblast cells for which predicted receptors NRP2, and MET are present on CTB. Together, they trigger proliferation and differentiation of CTB. In contrast, such interactions were not determined for STBs Developed on the basis of analysis from Vento-Tormo et al. (Vento-Tormo et al., 2018). **(B)** U-shaped trajectory from pseudo time analysis, where CTB is present at center while, EVT and STB are present at the opposite ends. STBs are further bifurcated one arm has upregulated hormone genes (CSH, GH, CGB) and the other arm showed upregulation of fusogens (ERVFRD-1, ERVW-1 ERVV1) Reproduced with permission from Tsang et al. (Tsang et al., 2017). TB, trophoblast; EVT, extra villous trophoblast cells; CTB, cytotrophoblast cells; STB, syncytiotrophoblast.

Similarly, Vento-Tormo et al. provided a comprehensive scRNA-seq data analysis of about 70,000 single cells from the maternal–fetal interface (11 deciduae and 5 placentas from samples obtained between 6 and 14 weeks of gestation) and 6 matched peripheral blood mononuclear cell samples (Vento-Tormo et al., 2018). Analysis of the TB cells revealed an expected and well-defined trajectory of CTB differentiation into STB and EVT. Vento-Tormo et al. also used their cell interaction repository CellPhoneDB (www.CellPhoneDB.org) of ligand–receptor interacting pairs and defined CTB that expressed many of the predicted ligands/receptors involved in the processes of proliferation and differentiation, e.g., epidermal growth factor receptor (EGFR)*,* placental growth factor (PGF) and hepatocyte growth factor (HGF). Also, as predicted, there was an upregulation on EVT of receptors involved in invasion, immunomodulation, and cellular adhesion whose ligands are produced by decidual cells. For instance, transcription of the gene encoding transforming growth factor-beta 1 (*TGFB1*) and its receptor increased as EVT differentiated. It is noteworthy here that TGFB1 functions to suppress immune responses (Mariathasan et al., 2018) and to activate epithelial–mesenchymal transitions. The potential identified ligand for ACKR2, which is a scavenger receptor of inflammatory cytokines, is TGFB1 produced by maternal immune cells (Figure 1A) (Madigan et al., 2010). Although Liu et al. comprehensively described many of the TB cell subpopulations and identified potential intercellular interactions, like others, they provided very little information concerning STB interactions with neighboring cells (Figure 1A). Some intriguing progress in the description of the human STB has been reported by Tsang et al. while constructing the TB trajectory from scRNA-seq data (Tsang et al., 2017). This analysis developed a well-formed and continuous u-shaped trajectory in which the STB and EVT are present at opposite ends and CTB present in the middle of the U, suggesting common or similar/related CTB progenitors. The STB end of this U-shaped trajectory is bifurcated with one arm showing a strong expression profile for genes involved in the cell fusion such as *ERVW-1* (*syncytin-1*), *ERVFRD-1* (*syncytin-2*), and *ERVV-1* while the other arm exhibits enrichment for genes driving the production of gestational hormones, *GH2* and *CGB* (Figure 1B) (Tsang et al., 2017). Although, scRNA-seq has provided detailed insight into TB subpopulations, the molecular signatures of these subtypes, interpopulation interactions at the molecular level, and trajectories from progenitor cells to more differentiated cells, our understanding of these processes in STB development has been limited using scRNA-seq because STB is very large in size, fragile and multi-nucleated and probably does not survive the processing that precedes library construction.

### snRNA-Seq and Syncytiotrophoblasts Transcriptome Profiling

Reproductive biologists have not been able to obtain and transcriptionally profile the TB of early human pregnancy, especially at the time when it invades into the uterine wall. This primitive TB exhibits a unique combination of a syncytialized morphology and invasive capacity and acquisition of primary samples of this primitive TB is severely restricted by a number of ethical and practical reasons. Consequently, three approaches are being established to mimic *in vivo* TB formation at such early stages of human gestation. The first involves extended embryo culture beyond the blastocyst stage and has been recently reviewed by Zhou et al. (Zhou et al., 2021). A second generates TB from human pluripotent stem cells (PSCs), and induced pluripotent stem cells (iPSCs) by exposing them to BMP4 and two small compounds, an activin A signaling inhibitor (A83-01) and a FGF2 signaling inhibitor (PD173074) and has been called the BAP model (Yabe et al., 2016; Roberts et al., 2018; Khan et al., 2021). The third approach uses TB stem cells (TSCs) and induced TSCs (iTSCs) (Oda et al., 2006; Roberts & Fisher, 2011; Dong et al., 2020). In a related approach, two groups have derived TSCs from naïve PSCs, i.e., human blastocysts and differentiated them successfully into different TB lineages (Okae et al., 2018; Dong et al., 2020). While scRNA-seq technologies have been used in many of these *in vitro* models of early human TB development, there remains limited transcriptomic data using this approach on STB development due to the limitations mentioned above.

**FIGURE 2:**
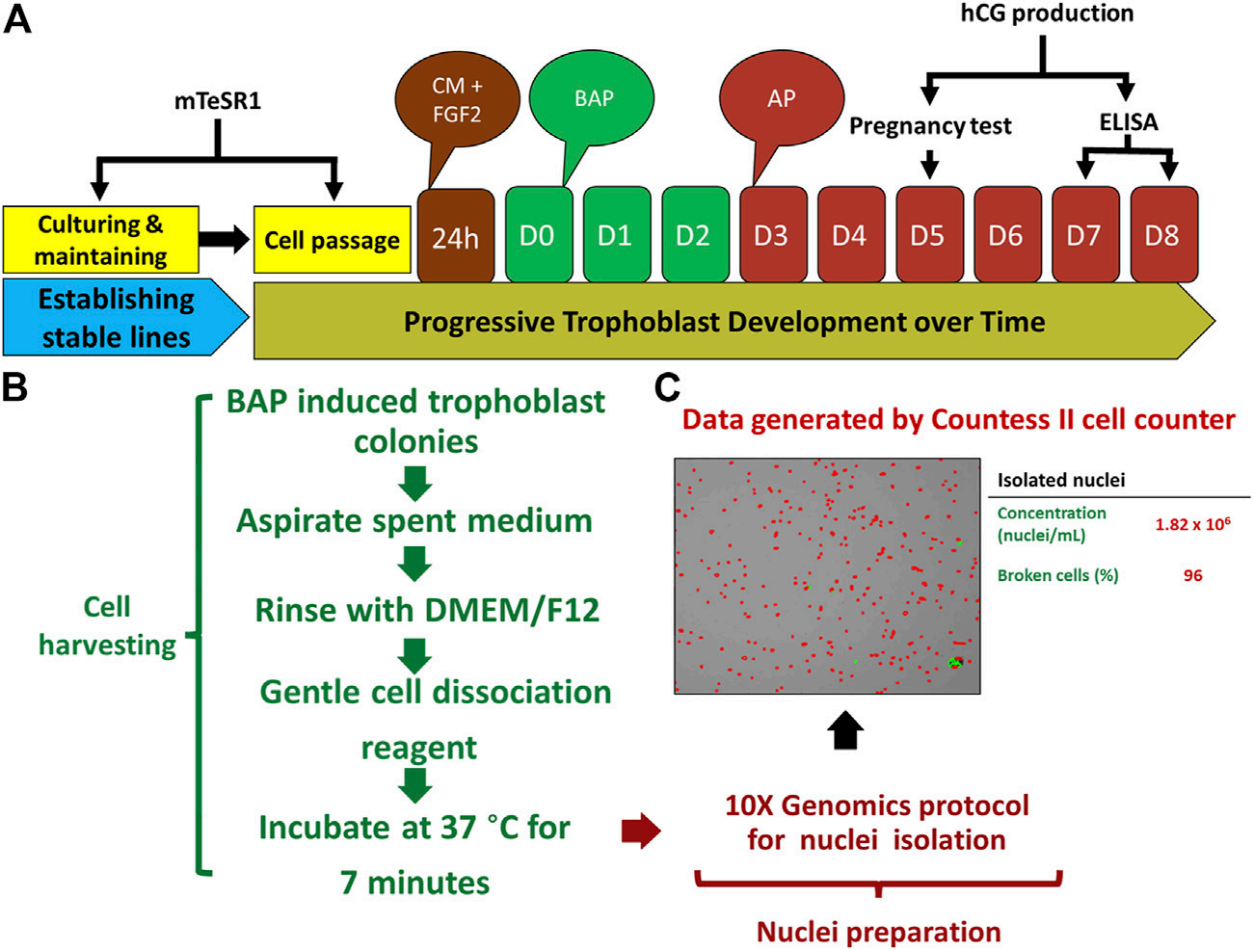
Protocol for TB development and nuclei isolation. **(A)** Strategy for BAP directed trophoblast development. Human embryonic stem cells (hESCs) thawed and passaged several times on mTeSR1 medium to established stable lines. The day following passaging, mTeSR1 medium was replaced with FGF2 (4 ng/μL) supplemented conditioned medium (mouse embryonic fibroblast (MEF)-conditioned DMEM/F12 with knockout serum replacement (KOSR) medium). For trophoblast (TB) induction, after 24 h, the cells were kept on DMEM/F12/KOSR (BAP/DMEM/F12/KOSR) medium supplemented with BMP4 (10 ng/ml), A83-01 (1 μM), and PD173074 (0.1 μM) for 3 days (BAP treatment), and the same medium without BMP4 (AP treatment) till day 8. **(B)** Strategy used for nuclei isolation. TB cell colonies were harvested by gentle cell dissociation reagent (GDR) (STEMCELL Technology, Cat# 07174) and were proceeded for nuclei isolation after 7 min of incubation at 37°C without aspirating GDR. **(C)** Representative data from one of isolated nuclei samples, left panel is the image showing isolated nuclei, right panel showed concentration of the nuclei and percentage of the broken cells from which the nuclei were collected. Reproduced with permission from Khan et al. (Khan et al., 2021). BMP4, Bone morphogenic protein-4; A, A83-01 (Activin/Nodal inhibitor); P, PD173074; BAP, BMP4 + A83-01 + PD173074; AP, A83-01 + PD173074; hCG, human chorionic gonadotropin; D, day.

**FIGURE 3:**
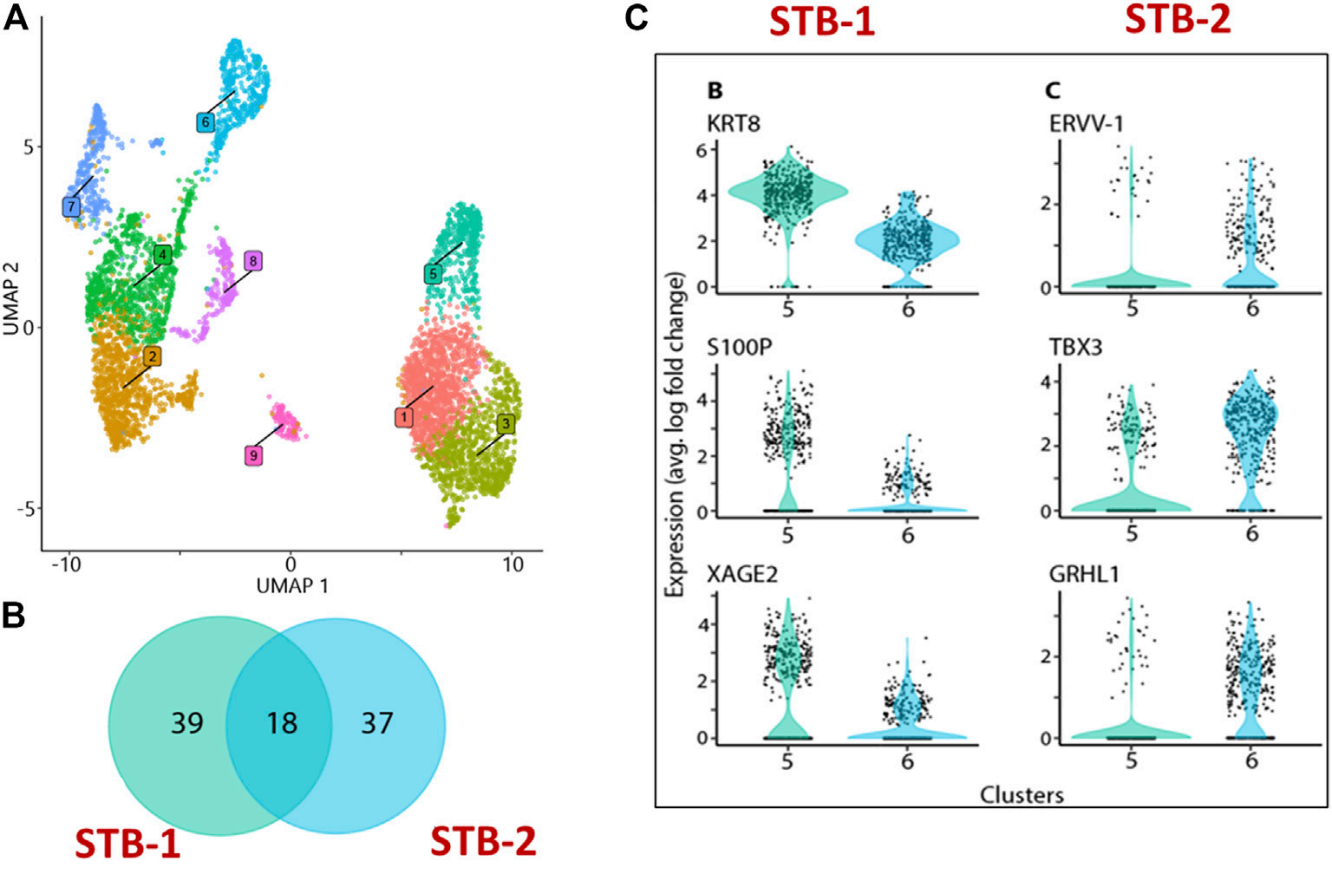
snRNA-seq from BAP directed TB. **(A)** Visualization of nuclei included in the analysis, colored according to assignment by clustering analysis. Five clusters: 1,4, 7, 8, and 9 were different kinds of CTBs, cluster 2 and 3 were EVT-like, and clusters 5 and 6 showed strong resemblance to human first trimester STB. **(B)** Details of differentially upregulated STB genes between STB-1 and STB-2. **(C)** Representative STB differentially upregulated genes showing expression across the nuclei from STB-1 (left panel) and STB-2 (right panel). STB-1 and STB-2 referring cluster 5 and 6 respectively from cluster analysis of snRNA-seq (Figure 3A). Reproduced with permission from Khan et al. (Khan et al., 2021). TB, trophoblast; EVT, extra villous trophoblast cells; CTB, cytotrophoblast cells; STB, syncytiotrophoblast.

Recognizing these limitations, we have recently employed snRNA-seq approaches to help to circumvent the cell size, cellular fragility and multinucleation challenges that STB presents to scRNA-seq approaches. Using snRNA-seq, we were able to generate a transcriptome profile for STB generated from human PSCs (H1) created by the BAP model (Khan et al., 2021). A two-step approach is commonly used to obtain nuclei from cells for use in snRNA-seq, in order: 1) harvest a cellular fraction with an optimized percentage of viable cells and 2) lyse these harvested cells to obtain a high proportion of intact nuclei. Typical two-step methods, however, are difficult in the large, multinucleated and fragile STB and the two steps were, therefore, merged in an effort to maximize the isolation of intact nuclei from living STB. Briefly, BAP-differentiated TB were cultured under 5% and 20% O_2_ conditions for 8 days to maximize STB formation (Figure 2A). Nuclei were then isolated according to the protocol described by 10X genomics (Genomics, 2021) without aspirating the cell harvesting reagent, gentle cell disassociation reagent (GDR) (Figures 2B,C). Using this approach, we were able to prepare nuclear suspensions comprised almost entirely of intact nuclei by lysing over 95% of the BAP-differentiated TB cells. Data generated on the 10X genomics platform from these samples (Stuart et al., 2019) distributed the isolated nuclei into nine clusters with two major groupings. One group was comprised of five clusters and the second of three clusters. One of the clusters appeared to be separated from the two main groups and was present only in cells that had been differentiated under low O_2_ conditions (Figure 3A). All of the clusters displayed well-described TB signatures such as *KRT7, GATA3,* and *TFAP2C* (Lee et al., 2016); four of these were different kinds of CTBs, two were EVT-like and two were highly enriched with STB markers, as determined by using a placental enrichment tool (Jain & Tuteja, 2021). The two STB clusters designated STB-1 and STB-2 (referring to cluster 5 and 6 respectively from snRNA-seq data; Figure 3A), displayed differential expression profiles for the genes considered to be STB signatures. The analysis identified at least 18 STB genes that were upregulated to approximately the same extent in both STB clusters, including the *CYP11A1* gene involved in progesterone production and *PGF*, 39 genes that were differentially upregulated in STB-1, including *KRT8, KRT1*8, *S100P*, *CGB8* and *XAGE2*, and 37 genes that were differentially upregulated in STB-2, including *ERVW-1*, *GULP1*, *ERVV-1,* and *TBX3* (Figures 3B,C). *CGA* (chorionic gonadotropin subunit A) was upregulated in STB-1 relative to STB-2 (expression values: STB-1 626.41 versus STB-2 130.94), but, surprisingly high levels of its transcripts were detected in all of the identified TB clusters and in almost all individual nuclei, including CTB and EVT, despite the fact that CGA protein can only be detected in STB and not throughout the cell colonies (Yabe et al., 2016). This may be a widespread phenomenon, i.e., proteins localized to STB, but transcripts also detected in other non-STB clusters. It should be recognized that the snRNA-seq approach delivers sequences of nuclear transcripts, which are largely unprocessed and possess regions transcribed from introns. Processing and other forms of editing may either select mRNA for transport out of the nucleus and for retention. These processes may be a mechanism of gene regulation (Bahar Halpern et al., 2015; Palazzo & Lee, 2018). Bulk RNA-seq and scRNA-seq are more likely to reflect the content of cytoplasmic mRNA and, in this sense, have an advantage over snRNA-seq.

Other differences distinguished STB-1 and STB-2. For example, when TFs implicated in TB differentiation were compared, more were upregulated in STB-2 (44 TFs) than in STB-1 (15 TFs). These differences are likely to reflect functional and developmental differences between the two STB clusters. We have compared our snRNA-seq transcriptome profiling of STB to published human placental scRNA-seq and show that the snRNA-seq can optimize the percentage of the analyzed components that are clustered as STB (Table 1). The snRNAseq approach identified and profiled a higher percentage (15.7%) of STB among collected cells compared to those studies using scRNA-seq. snRNA-seq proved a better tool than bulk and scRNA-seq for transcriptomic profiling of STB and should help to extend our understanding of this unique placental cell subtype. Further investigation that includes pseudo time analyses will be necessary to determine whether the two STB clusters identified by snRNA-seq are distinct or whether one acts as a progenitor (STB-2) for the other (STB-1), a scenario that we believe to be unlikely as nuclei with intermediate profiles are not detected.

**TABLE 1:** Comparison of STB percentage among samples analyzed by scRNA-seq or snRNA-seq.

	Sample types	Sample Size	Gestation (weeks)	Total cells/nuclei	STB cells/nuclei	STB Percentage	References
scRNA-seq	Placenta	7	8 and 24	1,567	64	4.08	Liu et al. (2018)
	Placenta	9	Term	77,906	174	0.22	Pique-Regi et al. (2019)
	Placenta	5	Term	20,518	N/A	4–5	Tsang et al. (2017)
	Placenta	2	Term	87	2	N/A	Pavlicev et al. (2017)
	Placenta and blood	12 + 6	6–14	70,235	Not provided		Vento-Tormo et al. (2018)
	Villi	8	6–11	14,341	1,104	7.7	Suryawanshi et al. (2018)
snRNA-seq	TB	4	BAP day 8	5,355	839	15.7	Khan et al. (2021)

STB, syncytiotrophoblast; scRNA-seq, single cell RNA sequencing; snRNA-seq, single nuclei RNA sequencing; TB, trophoblast; N/A, not applicable; BAP, BMP4 + A83-01 + PD173074.

## The Use of Stem-Cell Derived Trophoblast Models for Personalized Medicine: A Focus on Preeclampsia

An ultimate goal of our own on-going studies and that of the field as a whole is to develop disease-specific and personalized medicine approaches to disorders of human pregnancy, several of which clinically manifest only late in pregnancy. To do so, technologies such as those discussed earlier, e.g., model cell/organoid systems and scRNA-seq, need to be leveraged in appropriate model systems to allow discovery of underlying causal factors and functional mechanisms behind specific placental disorders. Of these diseases, PE is considered a main target as it is common and highly morbid for both mother and baby but its causes, prediction of disease in early pregnancy, and treatments remain enigmatic (Mol et al., 2016; Phipps et al., 2019). The only cure requires delivery of the baby and placenta, oftentimes compromising the health of the baby to minimize maternal morbidity and mortality. Adding to the difficulties in the study of preeclampsia are considerations of whether the disease is maternal or fetal/placental in origin (Roberts & Gammill, 2005; Steegers et al., 2010) and whether the pathogenesis differs between common subgroupings of PE (e.g., early vs. late-onset; see below) (Ogge et al., 2011). There is little controversy, however that PE is inextricably linked to abnormal placentation, including insufficient EVT invasion and defects in syncytial fusion and apoptosis (Chaddha et al., 2004; Langbein et al., 2008; Khong et al., 2015). To study such disease subgroup- and possibly even patient-specific features of PE, patient-derived *in vitro* TB models are needed but still incompletely developed (Sheridan et al., 2019; Horii et al., 2021). Recently, Okae et al. established a culture system of human blastocyst- and first trimester placenta-derived TSCs which have self-renewal ability and can give rise specifically to either STB or EVT, depending on the medium used (Okae et al., 2018). Combined with other stem-cell derived TB models, we are entering a new era in the study of PE that employs TSC culture systems and patient-generated cell sources.

### Trophoblast Sublineage Abnormalities in PE

PE has often been subcategorized into two different, albeit related disease entities: 1) early-onset PE (EOPE) which develops before 34 weeks of gestation and 2) late-onset PE (LOPE) which develops after 34 weeks of gestation (Valensise et al., 2008). While both have similar characteristic maternal signs and symptoms, EOPE is more severe and is much more closely linked to fetal growth restriction (FGR) and adverse maternal and neonatal outcomes. Defects in both TB invasion and in STB formation and turnover have been linked to EOPE, although the former is much better studied and may be causal of some of the defects seen in the latter. Several lines of evidence point to shallow invasion of EVT into the maternal decidua and subsequent deficient remodeling of the uterine spiral arteries during early pregnancy as causal in EOPE (Say et al., 2014; Khong et al., 2016). These changes, in turn, are thought to cause placental malperfusion characterized by high blood pressure/low blood flow to the placenta, poor spiral artery reactivity to changes in oxygen tension and episodes of placental hypoxia and hyperoxia that cause placental oxidative damage (Romero & Chaiworapongsa, 2013; Hansson et al., 2014; San Juan-Reyes et al., 2020) The relatively high placental perfusion pressure can erode the surface of the outer STB layer of the chorionic villi. As a result, there is excessive detachment and release of STB fragments, including microparticles, extracellular vesicles and apoptotic bodies, into the maternal blood circulation. Additionally, there is increased release of anti-angiogenic factors, such as soluble fms-like tyrosine kinase 1 (sFLT-1) and soluble endoglin (sENG) as well as pro-inflammatory molecules, including damage-associated molecular patterns (DAMPs) (Wang et al., 2019; Rana et al., 2020). These anti-angiogenic factors, sFLT-1 and sENG play roles as decoy receptors, trapping major essential pro-angiogenic factors, such as PGF, vascular endothelial growth factor (VEGF) and TGF-β1, and thereby making them unavailable for normal physiological homeostasis. This underlying pathophysiologic cascade is associated with the defective maternal angiogenesis and endothelial dysfunction observed in EOPE syndrome (Levine et al., 2004; Levine et al., 2006; Gregory et al., 2014). There do, however, appear to be primary defects in STB formation in the PE placenta. For instance, mRNA and protein levels of the profusogenic human endogenous retroviral envelope gene product, syncytin-1, are reduced in PE placentas (Vargas et al., 2011; Ruebner et al., 2013; Zhuang et al., 2014). Syncytin-1 is felt to be central to TB fusion and STB turnover, and diminishment in STB turnover inhibits the renewal of the stressed PE STB that is releasing bioactive molecules into the maternal circulation. This can create a feed-forward loop that results in persistence and worsening of EOPE symptoms. When placental syncytin-1 expression was compared among the monozygotic dichorionic twins with birth weight discordance and singleton normal-weight controls, syncytin-1 levels were inversely correlated with birth weight (Gao et al., 2012). This suggests that defective STB development and/or turnover can hinder fetal growth and fetal growth restriction is common in EOPE. The precise mechanisms underlying impaired syncytialization in EOPE, with or without fetal growth restriction, remain understudied and unclear.

### Limitations to *In vitro* Study of EOPE

Despite hundreds of years of study, much remains to be learned about basic disease mechanisms leading to EOPE and almost nothing has been discovered to allow for early diagnosis or prevention of this common and potentially devastating disease. Central to these difficulties is the fact that EOPE underpinnings likely arise during very early placental development, but the disease does not manifest clinically until much later in pregnancy (Roberts et al., 2017). Couple this with ethical and logistical limitations to the study of primary clinical samples during ongoing pregnancies, not the least of which is the robust preclinical development of human pregnancy that occurs prior to documentation of a positive pregnancy test, and the need for *in vitro* models to study this disorder becomes unmistakable. Many of the existing models, however, have specific drawbacks. Primary TB cells extracted from second and third trimester human placentas after delivery, which can be gathered after PE diagnosis, are not only short-lived, but have already undergone lineage-specific differentiation and therefore cannot be used in the study of abnormalities in lineage development that may be causal in the disease (Ringler & Strauss, 1990). Commonly available rodent models insufficiently mimic many important morphological and functional characteristics of human placental development that may be central to PE pathogenesis (Pennington et al., 2012; Roberts et al., 2018). Burgeoning advances in cellular and molecular technologies; however, have enabled us to acquire patient- and disease-specific human iPSCs with a capacity for unlimited self-renewal and differentiation into any cell type of interest (Takahashi et al., 2007; Lujan et al., 2012), including human placental cells. This has allowed, for the first time, the derivation of *in vitro* models that can leverage cells and tissues obtained after PE disease onset to study early stages of placental development and sub-lineage differentiation, an approach that is both disease-specific and personalized.

### Modeling EOPE Trophoblast Through BAP or BMP4 Treatment of Induced Pluripotent Stem Cell

In order to establish an EOPE model, Sheridan et al. generated iPSCs by reprogramming fibroblasts extracted from the umbilical cords of placentas delivered by mothers with and without EOPE (Sheridan et al., 2019). These iPSC lines were converted into TB under the BAP protocol described above. When compared to BAP-treated TB cells from unaffected mothers, those derived from pregnancies affected by EOPE had defects in their capacity to invade a surrounding substratum. Such invasion defects are a hallmark of EOPE placentation *in vivo* (Pijnenborg et al., 2011; Khong et al., 2015). These *in vitro* invasion defects were oxygen-sensitive and manifested in hyperoxic (20% O_2_) but not normoxic (5% O_2_) conditions. Supporting previous reports that low O_2_ conditions (2%) suppressed PGF production (Fujii et al., 2017), PGF secretion increased in control TB under 20% O_2_ compared with 5% O_2_ whereas that in EOPE TB showed no significant response to O_2_ conditions, suggesting poor physiologic response to changes of oxygen tension in EOPE TB. As PGF is primarily produced and secreted by STB, these findings further demonstrate that placental abnormalities associated with PE can affect multiple TB lineages. More recently, Horii et al. also established an EOPE TB model from iPSCs generated from umbilical cord-derived mesenchymal stem cells (MSCs) (Horii et al., 2021). To induce TB differentiation, the authors cultured these iPSCs using conditions that were similar to those used by Sheridan et al., but that differed in potentially important aspects. Hori et al. differentiated their cells in irradiated mouse embryonic fibroblasts-conditioned medium (MEF-CM) supplemented with BMP4 while Sheridan et al. induced TB differentiation using BAP without concurrent exposure to MEF-CM, although the cells were exposed to MEF-CM for a day prior to BAP treatment. Using the former conditions, although Hori, et al. (Horii et al., 2021) reported no difference between control TB and EOPE TB in their capacity to invade in either of their O_2_ conditions (2% and 21%), EOPE TB did display blunted changes in invasion and secretory hCG production when switched to low oxygen conditions (2%) when compared to control TB. Taken together, these findings suggest that the patient-specific iPSC-derived EOPE TB model can mimic known characteristics of EOPE placental dysfunction, including abnormal responses to oxidative stress and that STB and EVT can each display measurable abnormalities.

### Retention of Epigenetic Memory During Reprogramming

Considering the EOPE-like features revealed in the patient-specific iPSC-derived TB models (Sheridan et al., 2019; Horii et al., 2021), it is plausible that the epigenetic marks of EOPE are inherited from parental cells. That iPSCs can retain donor-specific epigenetic characteristics has been demonstrated in several systems (Kim et al., 2011; Shao et al., 2013; Noguchi et al., 2018). More specifically, a comparison of DNA methylation profiles among discrete individual iPSCs and their parental clones revealed that iPSC clones generated from the same patient had very similar methylation profiles, whereas the cells from individual patients exhibited interindividual differences, especially in non-promoter regions and outside of CpG islands (Shao et al., 2013). In the iPSCs generated from umbilical cord derived MSCs, DNA methylation patterns were also maintained when comparing the original MSCs to daughter iPSCs (Horii et al., 2021). It is further notable that the fibroblasts extracted directly from the control and EOPE umbilical cords displayed EOPE-specific oxygen sensitivities characterized by a marked stress response to acute O_2_ changes (Yang et al., 2014). Further, while the daughter iPSCs lost this differential sensitivity, the TB generated from these iPSCs again displayed EOPE-specific oxygen sensitivity with defective responses in EOPE TB but not control TB under hyperoxic conditions (Sheridan et al., 2019). This suggests that the oxygen sensitivity of the parent fibroblasts was not merely a reflection of their environment and health at the time of delivery and cell isolation but was instead representative of their epigenetic status. Further, this epigenetic status could be preserved and manifested when daughter iPSCs were differentiated into TB (epigenetic memory) (Sheridan et al., 2019). Reprogramming of patient- and disease-specific umbilical cord-derived mesenchymal cells for TB generation may signify a promising means to investigate etiological genetic and epigenetic features of EOPE.

### Future Approaches for iTSC-Derived EOPE Modeling

Although the iPSC-derived EOPE TB models involving differentiation under BAP or BMP4 mentioned above have demonstrated disease-specific susceptibility to environmental stressors, more specific information on the functional mechanisms and pathophysiologic pathways underlying EOPE was not gleaned from these studies (Sheridan et al., 2019; Horii et al., 2021). Both models failed to identify significant differently expressed gene sets when comparing control and EOPE samples by bulk RNA-seq analyses. Because these models contain a randomly mixed population of CTB-, STB-, and EVT-like cells, specific details of lineage- and stage-specific TB differentiation features may be obfuscated. Complicating these analyses further is the likely underrepresentation of STB transcripts in these mixed TB lineage analyses due to the limitations mentioned in part one of this review. In addition, since EOPE is almost certainly multifactorial and its manifestations likely have a common pathway for several possibly distinct but converging defects in the processes of EVT and/or STB development (e.g. deficient CTB fusion, high STB apoptotic rate, and shallow EVT invasion) (Chaddha et al., 2004; Langbein et al., 2008; Khong et al., 2015), further definition of this disorder requires TB lineage-specific models.

Recently, a culture system for human TSCs derived from blastocysts and first-trimester placental CTB was successfully established by Okae et al. (Okae et al., 2018). These human TSCs displayed requisite endless self-renewal but also the capacity for lineage-specific differentiation into STB and EVT. When human TSCs are exposed to forskolin (a cAMP agonist) in the absence of Wnt activators and TGFβ1 inhibitors, the cells aggregate and fuse to form large syncytia (Okae et al., 2018), mimicking STB. In the presence of Matrigel, NRG1, and A83-01 (TGFβ1 inhibitor), on the other hand, TSCs differentiate toward spindle-shaped EVT-like cells characterized by high HLA-G expression and an invasion phenotype. Transcriptome analyses have shown that TSC-derived STB and EVT gene expression patterns very closely approximate primary STB and EVT, respectively. Moreover, engraftment of TSCs into immunodeficient mice induced the expression of representative placental marker genes including *ITGA6, KRT7, SDC1*, and *HLA-G* in dermal and subcutaneous tissues and high serum hCG levels, further promoting the value of TSCs as an *in vitro* model for human TB development.

The original TSCs described by Okae et al., however, were neither patient-specific nor could they model diseases such as EOPE that clinically manifest late in pregnancy. In response to these limitations, placental researchers, including those interested in EOPE, have begun to combine TSC and iPSC technologies (Li et al., 2019; Castel et al., 2020; Dong et al., 2020; Guo et al., 2021; Io et al., 2021; Mischler et al., 2021; Wei et al., 2021) and have even begun to generate TSCs directly from human fibroblasts (Liu et al., 2020). Although the methods have yet to be compared, so it remains unclear which is superior for the generation of *bona fide* TSCs, these methods should ultimately provide necessary pregnancy disease specific *in vitro* TSC models. Once TSC conversion in these models has been optimized, their capacity for lineage-specific differentiation can be leveraged to study TB lineage-specific contributions to diseases such as PE. Using these emerging technologies, our laboratory is developing an EOPE iTSC model through direct conversion from control- and EOPE-derived umbilical cord-fibroblasts through use of episomal plasmid vectors for reprogramming. We will use this model to identify differences in lineage-specific features of normal and abnormal TB proliferation, differentiation, invasion, secretory capacity and cell cycling between control and EOPE lines. In addition, we are using these cells to develop a self-renewing TSC organoid culture system that mimics the villous placenta and allows for the study of cell subtype interactions in three dimensions (Sheridan et al., 2021). In combination with the advances outlined in part one of this review, we also plan to apply snRNA-seq analyses to the iTSC-derived EOPE models, with a specific goal of improving our understanding of the STB functional phenotype. We expect application of snRNA-seq technology to EOPE-iTSC models will provide a more comprehensive understanding of the pathophysiology of EOPE, allowing for long-sought advances in disease diagnosis, prevention and treatment.
